# Study of Heat Transfer Characteristics of PCMs Melting Inside Aluminum Foams

**DOI:** 10.3390/ma18225130

**Published:** 2025-11-11

**Authors:** Farjad Shahid Hasan Khan, Andrea Diani

**Affiliations:** Department of Industrial Engineering, University of Padova, via Venezia 1, 35131 Padova, Italy; farjadshahidhasan.khan@studenti.unipd.it

**Keywords:** phase change materials (PCMs), T-history method, pores per inch (PPI), empirical modeling, metal foam composites, heat flux variation, thermal energy storage (TES)

## Abstract

This study examines the thermal performance of phase change material (PCM)–metal foam composites under base heating, a configuration more relevant to compact thermal energy storage (TES) and electronics-cooling applications, compared to the widely studied side-heated case. Metal foams with pore densities of 10, 20, and 40 PPI, but identical porosity (volumetric value), were impregnated with two PCMs (paraffin RT55 and RT64HC) and tested under varying heat fluxes. The thermophysical properties of three PCMs (RT42, RT55, and RT64HC) were first characterized using the T-history method. A control case consisting of pure PCM revealed significant thermal lag between the heater and the PCM, whereas the inclusion of a metal foam improved temperature uniformity and accelerated melting. The results showed that PPI variation had little influence on melting completion time, while PCM type, viz., melting temperature, strongly affected duration. Heat flux was the dominant parameter: higher input power substantially reduced melting times, although diminishing returns were observed at elevated heat fluxes. An empirical correlation from the literature, originally developed for side-heated foams, was applied to the base-heated configuration and reproduced the main melting trends, though it consistently underpredicted completion times at high fluxes. Overall, embedding PCMs in metal foams enhances heat transfer, mitigates localized overheating, and enables more compact and efficient TES systems. Future work should focus on developing correlations for non-adiabatic cases, exploring advanced foam architecture, and scaling the approach for practical energy storage and cooling applications.

## 1. Introduction

The growing integration of renewable energy technologies and the increasing demand for efficient thermal management solutions have intensified research into advanced thermal energy storage (TES) systems. Among the available options, latent heat storage using phase change materials (PCMs) has emerged as one of the most promising approaches due to its high energy density, nearly isothermal operation, and potential to smooth mismatches between supply and demand [1,2,3]. These features enable PCMs to play a vital role in applications such as building heating and cooling [4], battery and electronics thermal regulation [5], and renewable energy storage and conversion [6,7].

However, the low intrinsic thermal conductivity of most PCMs (<0.5 W·m^−1^·K^−1^) severely restricts their heat transfer rates, leading to slow charging/discharging and significant thermal gradients within storage media [8,9]. Over the past two decades, several enhancement strategies have been proposed, including nanoparticle addition [10], micro- and nano-encapsulation [11,12], and the incorporation of highly conductive porous matrices [13,14]. Among these, embedding PCMs in metallic foams has been identified as particularly effective, as the interconnected ligaments provide conductive pathways that enhance heat transfer while retaining large latent storage capacity [15,16,17].

A number of experimental and numerical studies have demonstrated the benefits of PCM–metal composites. Zhang et al. [11] and Zhao and Wu [13] reported accelerated melting in PCM composites with expanded graphite and graphite foams, while Lafdi et al. [14] and Zhang et al. [15] highlighted their suitability in building-integrated TES. Youssef et al. [16] and Wang et al. [17] studied PCM–metal foams and found that thermal response is strongly influenced by pore structure and boundary conditions. Diani and Campanale [18] provided detailed experimental evidence that paraffin waxes embedded in aluminum foams show significantly improved transient melting performance compared to bulk PCMs. Parallel advances in additive manufacturing now make it possible to fabricate metal foams with highly controlled architectures [19], opening pathways to optimize geometry and tailor PCM–metal interactions.

Equally important for TES design is the accurate characterization of PCM thermophysical properties. While differential scanning calorimetry (DSC) is commonly used, its reliance on milligram-scale samples may fail to capture the behavior of bulk or heterogeneous PCMs [20]. To overcome this limitation, Rady et al. [20] applied the T-history method to granular composites, enabling derivation of enthalpy–temperature relationships more representative of real operation. More recently, Ayora-Fernández et al. [21] optimized the T-history method for heterogeneous PCMs such as emulsions and nano-suspensions, while Zhou et al. [22] further validated its robustness as a practical, cost-effective alternative to DSC.

Despite these advances, research on PCM–metal foam composites remains limited, particularly under *base-heated* configurations. Most prior work, including Diani and Campanale [18], has focused on side-heated foams, whereas base heating is more relevant to applications such as electronics cooling, compact TES modules, and surface-integrated energy systems. The difference in heating geometry can fundamentally alter melting dynamics, thermal gradients, and heat loss behavior, yet this has not been systematically studied.

Very recent literature (2024–2025) reinforces these points and adds nuance. A comprehensive 2024 review synthesizes latent-heat TES with metal foams, noting strong gains in charging rates but also the trade-off with reduced effective storage density and the need to tailor foam architecture to operating conditions [23]. In electronics-cooling contexts, 2024 experiments on partially filled PCM heat sinks with metal foam identified an optimal foam/PCM fill ratio that maximizes thermal performance while limiting mass and losses—evidence that geometry and non-fully filled configurations can be advantageous for compact devices [24]. Complementing this, a 2025 study systematically varied foam porosity and PPI, showing that lower porosity (higher solid fraction) foams substantially accelerate melting but at the expected penalty of energy density—underscoring the design trade-off space relevant to the base-heated modules [25]. These recent findings motivate the present study’s focus on base heating and controlled foam architectures as levers for practical TES and electronics-cooling applications.

The present work addresses this gap by experimentally investigating PCM–metal foam composites subjected to base heating. Specifically, the effects of pore-per-inch (PPI) density (10, 20, 40), PCM type (RT55, RT64HC), and applied heat flux (6.25, 12.50, and 18.75 kW m^−2^) on thermal performance are analyzed. The T-history method is first used to characterize PCM (RT42, RT55 and RT64HC) properties, ensuring reliable input data for analysis. The experimental results are then compared with the empirical correlation of Diani and Campanale [18], highlighting the influence of heating geometry and external heat losses. This study provides new insights into the design of PCM–metal foam composites for high-performance TES and thermal management applications.

## 2. T-History Method

Before studying the PCM–metal composites, it was necessary to verify the thermal properties of the PCMs. For this study, the paraffins RT42, RT55, and RT64HC were used. According to the values reported by the supplier (Rubitherm, Berlin, Germany), the specific heat capacity of all PCMs was given as 2 kJ·kg^−1^·K^−1^, regardless of type. To obtain more reliable data, the T-history method was employed, as it represents a simpler and more economical alternative to differential scanning calorimetry (DSC) [22].

In this method, the PCM is first melted inside a container of known specific heat capacity (*c_pt_*) and mass (*m_t_*). In our study, stainless steel containers were used, while water was placed in a separate container of identical dimensions and material to serve as the reference. Both containers were heated to the same initial temperature, below the boiling point of water, and then allowed to cool naturally to ambient conditions without external heating or cooling, in order to guarantee a low value of heat transfer coefficient on the air side.

To ensure the validity of the T-history assumptions, two requirements must be satisfied. First, the Biot number should be less than 0.1, so that the temperature distribution within the sample can be considered uniform and the lumped capacitance model can be applied. Second, the ratio of length to diameter of the tube should be greater than 10 to guarantee that radial heat transfer dominates over axial conduction [20]. The current setup satisfies both the requirements.

**Figure 1:**
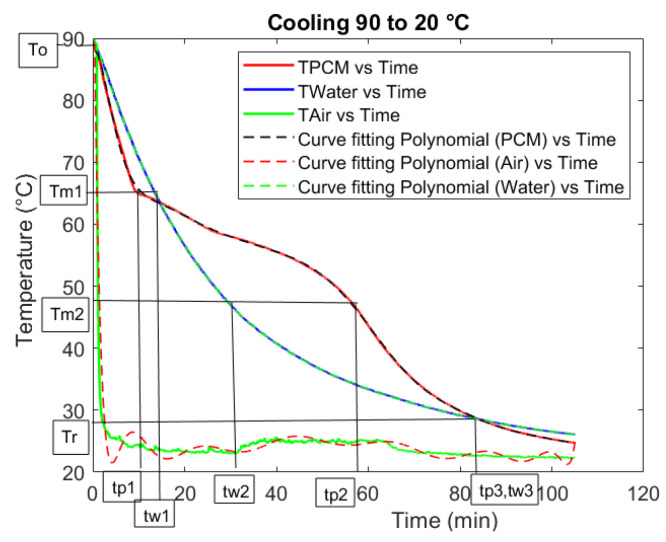
Results of a T-history test (RT64HC).

In Figure 1 (with Table 1, showing the meanings of the symbols on the ordinate axis), the temperature vs. time graph of a T-history test is shown. This test was for Rubitherm RT64HC. For the calculation of the specific heat of solid (*c_ps_*) and liquid (*c_pl_*) PCM, it was necessary to find the time associated with the start and end of the phase change (*t_p_*_1_ and *t_p_*_2_ for the PCM, and *t_w_*_1_ and *t_w_*_2_ for water), and with the common end temperature for both the PCM and water after the PCM phase change completion (*t_p_*_3_ for the PCM, and *t_w_*_3_ for water). In this case, the *T_r_* is selected such that *t_p_*_3_ and *t_w_*_3_ coincide.

Now, we need to use energy balance to make the equations that will be used to calculate the specific heats.

### 2.1. Liquid to the Beginning of the Phase Change

Energy released by the liquid PCM and the container until the start of the phase change is equal to the energy gained by the ambient (with temperature *T_a_*) in contact with the outside surface of the container (with an area *A_t_*).

(1) $$\left(m_{t}c_{pt}+m_{p}c_{pl}\right)\left(T_{0}-T_{m1}\right)=hA_{t}\int_{0}^{t_{p1}}\left(T-T_{a}\right)dt=hA_{t}A_{p1}$$

In Equation (1), *m_p_* and *c_pl_* are the mass and specific heat of the liquid PCM, *m_t_* and *c_pt_* are the mass and specific heat of the container, *T*_0_ is the initial homogeneous temperature, *T_m_*_1_ and *t_p_*_1_ are the temperature and time at which the phase change starts, and *A_p_*_1_ is the area between the PCM and the ambient air temperature profiles in the considered time frame. Plus, *h* is the natural convection coefficient of air.

For reference fluid, i.e., water, the following equation is formulated.

(2) $$\left(m_{t}c_{pt}+m_{w}c_{pw}\right)\left(T_{0}-T_{m1}\right)=hA_{t}\int_{0}^{t_{w1}}\left(T-T_{a}\right)dt=hA_{t}A_{w1}$$

where *m_w_* and *c_pw_* are the mass and specific heat of water inside the container, *t_w_*_1_ is the time corresponding to the temperature *T_m_*_1_ achieved by the reference fluid (water) and *A_w_*_1_ is the area between the water and ambient air temperature profiles in the considered time.

These two equations are solved simultaneously to get the following relationship.

(3) $$c_{pl}=\frac{\left(m_{t}c_{pt}+m_{w}c_{pw}\right)}{m_{p}}\frac{A_{p1}}{A_{w1}}-\frac{m_{t}c_{pt}}{m_{p}}$$

### 2.2. Beginning of the Phase Change to the End of the Phase Change

The energy released by the liquid PCM and the container during the phase change process is equal to the energy gained by the ambient air (with temperature *T_a_*) in contact with the outside surface of the container (with an area *A_t_*).

(4) $$\left(m_{t}c_{pt}+m_{p}c_{pm}\right)\left(T_{m1}-T_{m2}\right)+m_{p}H_{l}=hA_{t}\int_{t_{p1}}^{t_{p2}}\left(T-T_{a}\right)dt=hA_{t}A_{p2}$$

*c_pm_* is the specific heat of the PCM during the phase change, as some contribution of sensible heat is observed during the PCM phase change. *T_m_*_2_ and t*_p_*_2_ are the temperature and time at the end of the phase change process. This coefficient can be approximated as the average of the *c_pl_* and *c_ps_*.

*H_l_* is the latent heat of the PCM. Similar equations can be formed for water.

(5) $$\left(m_{t}c_{pt}+m_{w}c_{pw}\right)\left(T_{m1}-T_{m2}\right)=hA_{t}\int_{t_{w1}}^{t_{w2}}\left(T-T_{a}\right)dt=hA_{t}A_{w2}$$

Simultaneous solution of these two equations gives the following relationship.

(6) $$H_{l}=\frac{\left(m_{t}c_{pt}+m_{w}c_{pw})(T_{m1}-T_{m2}\right)}{m_{p}}\frac{A_{p2}}{A_{w2}}-\frac{\left(m_{t}c_{pt}\right)\left(T_{m1}-T_{m2}\right)}{m_{p}}-c_{pm}(T_{m1}-T_{m2})$$

### 2.3. End of the Phase Change to the Common End Temperature

The following equations are like Equations (1)–(3).

(7) $$\left(m_{t}c_{pt}+m_{p}c_{ps}\right)\left(T_{m2}-T_{r}\right)=hA_{t}\int_{t_{p2}}^{t_{p3}}\left(T-T_{a}\right)dt=hA_{t}A_{p3}$$

*T_r_* is the common temperature at the end of the phase change. *t_p_*_3_ is the time for the PCM when it reaches temperature *T_r_*. *A_p_*_3_ is the area between the PCM and ambient air temperature profiles in the considered interval.

(8) $$\left(m_{t}c_{pt}+m_{w}c_{pw}\right)\left(T_{m2}-T_{r}\right)=hA_{t}\int_{t_{w2}}^{t_{w3}}\left(T-T_{a}\right)dt=hA_{t}A_{w3}$$

*t_w_*_3_ is the time when water reaches temperature *T_r_*. *A_w_*_3_ is the area between the water and ambient air temperature profiles in the considered interval.

Simultaneous solutions of Equations (7) and (8) yield the following relationship.

(9) $$c_{ps}=\frac{\left(m_{t}c_{pt}+m_{w}c_{pw}\right)}{m_{p}}\frac{A_{p3}}{A_{w3}}-\frac{m_{t}c_{pt}}{m_{p}}$$

**Figure 2:**
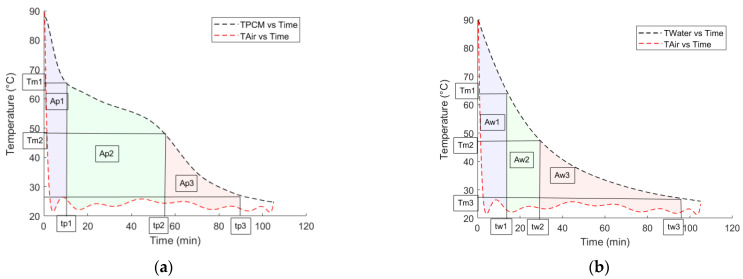
(**a**,**b**) represent the temperature–time profiles obtained using the T-history method for the paraffin RT64HC and the reference fluid (water), respectively. In both cases, the temperature variation of the samples and the ambient air is plotted over time during the cooling process. The shaded areas (*A_p_*_1_, *A_p_*_2_, *A_p_*_3_ for the PCM and *A_w_*_1_, *A_w_*_2_, *A_w_*_3_ for water) represent the energy exchange regions used to calculate the specific heats and latent heat. The characteristic time points (*t_p_*_1_, *t_p_*_2_, *t_p_*_3_ for the PCM and *t_w_*_1_, *t_w_*_2_, *t_w_*_3_ for water) correspond to the start, end, and completion of the phase change process, while *T_m_*_1_ and *T_m_*_2_ indicate the onset and completion temperatures of melting.

Figure 2, below, shows how the results of the integrals from the aforementioned equations would look on a temperature–time graph.

### 2.4. Results of the T-History Method

Using the data acquired by the experiments for the paraffins RT42, RT55, and RT64HC, the results reported in Table 2 were obtained. The melting range was defined as the points farthest from the melting process, because when melting starts, spikes in *c_p_* can be observed during solidification and liquefaction. Doing so resulted in more uniform results, less prone to abrupt changes when the range was altered slightly. These results were compared with the values reported by Rubitherm in the associated datasheets for each PCM.

## 3. Experimental Setup and Methodology

**Figure 3:**
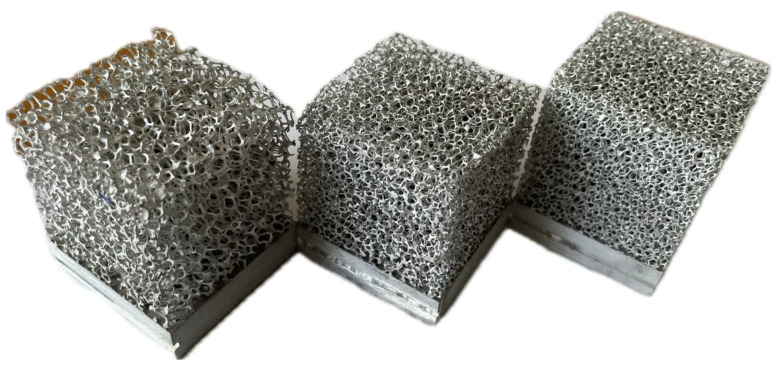
PPI 10, PPI 20, and PPI 40 (from left to right). Aluminum metal foams procured from *ERG Aerospace Corporation*. The manufacturer only supplies aluminum foams of PPI 5, 10, 20, and 40. Only non-metal foams are available in higher PPIs. Image captured in the lab.

After the T-history method, and the fabrication of the metal foams (all with the dimensions 4 × 4 × 4 cm^3^) with the same porosity but different pore density (pores per inch (PPI)), as depicted in Figure 3, the following parameters were considered:

A. Effect of the PPI variation on the phase change completion time, while all other variables are kept constant.
B. Effect of the PCM variation on the phase change completion time, while all other variables are kept constant.
C. Effect of the heat flux variation on the phase change completion time, while all other variables are kept constant.

The metal foams, with the main properties as shown in Table 3 below, were encapsulated on four sides (excluding the top) using polycarbonate sheets and high-temperature silica gel, after which they were filled with the PCM under investigation. The base of the metal foam was heated using an electric heater, with thermal paste applied at the interface to ensure uniform heat transfer. Temperature measurements were obtained using thermocouples: one junction was positioned at the mid-point of the foam, while another was inserted at the base in direct contact with the heater. Both thermocouples (having an accuracy of ±0.5 K, previously calibrated with a Pt100 as reference temperature sensor) were connected to the zero-point reference junction, and their data was fed to the data acquisition system.

For comparison, a control experiment was conducted using an identical container filled only with PCM, without a metal foam. This allowed evaluation of the influence of the foam presence and porosity on the thermal response of the system.

**Figure 4:**
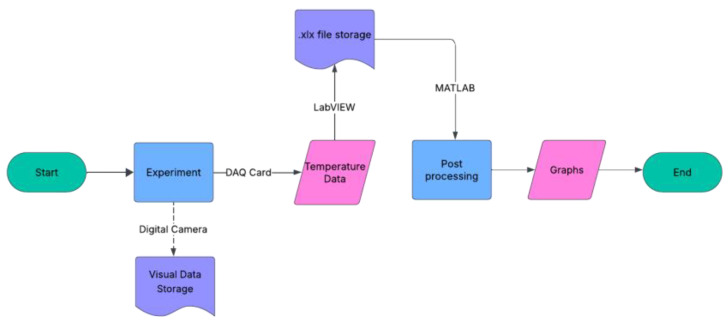
Simplified workflow of the experimental methodology. The process starts with the experiment, where temperature and visual data are acquired simultaneously. Temperature data are stored in .xlsx files for post-processing and analysis, while visual data are saved separately. Processed data are used to generate graphical results for interpretation. The experiment is declared to be completed after the visual inspection of the apparatus, i.e., when all the paraffin seems to be melted.

Data acquisition was carried out using a data acquisition (DAQ) system NI cDAQ-9178 equipped with NI-9213 modules for thermocouples and an NI-9219 module that is a universal module used for voltage measurements. The DAQ system digitized the thermocouple signals and transferred them to a computer via LabVIEW. The acquired data were stored in files and further processed in MATLAB R2023b—academic use to generate plots of the studied parameters. In parallel, a digital camera was employed to capture images of the test apparatus at 20 s intervals. These images were compiled into videos that supported the interpretation of thermal behavior, for example by correlating slope changes in the temperature–time curve with the onset of PCM melting observed visually. The flowchart of the methodology for the experimental tests is reported in Figure 4.

**Figure 5:**
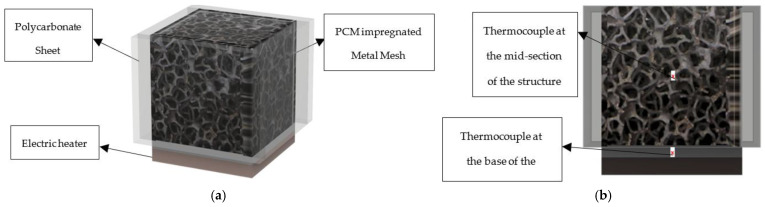
Experimental setup for PCM–metal mesh composites: (**a**) schematic of the test assembly showing the PCM-impregnated metal mesh encapsulated within polycarbonate sheets and heated from the base by an electric heater; (**b**) cross-sectional view indicating thermocouple positions at the mid-section of the mesh and at the base in contact with the heater. These sensors were used to monitor internal and boundary temperatures during melting.

For each reading, the PCM–metal composites are subjected to a heat flux, and the temperature of the base plus the mid-point of the metal composite is measured every second. The heat flow rate varied, in steps of 10 W, from 10 W to 30 W, to which heat fluxes of 6.25, 12.50 and 18.75 kW m^−2^ correspond, and the metal foams had pore densities of 10, 20, and 40 PPI. All combinations of the enclosure (empty and with the metal foams), heat flow rate, and pore densities were studied. Figure 5, shows the CAD drawing of the PCM-impregnated metal mesh, the heater and sensor positions. Data were used to generate graphs to fulfill the objectives of this experiment.

The electrical heater is connected to a stabilized DC power supplier. The heat flow rate is measured as the product between the current *I* flowing in the electrical heater and the voltage drop Δ*V_heater_* across it. Δ*V_heater_* is directly measured. The current is evaluated as the ratio between a voltage drop (Δ*V_shunt_*) measurement across a calibrated reference electrical resistance (shunt) and the known value of the resistance of the shunt itself (*R_shunt_*). The shunt is in series with the electrical heater. With this technique, the heat flow rate is evaluated within ±0.13% of the reading.

## 4. Results

The thermal performance of PCM–metal foam composites was evaluated with respect to three main variables: (A) pore density (PPI), (B) melting temperature of the PCM, and (C) applied heat flux. In all experiments, phase change completion time was selected as the main indicator of performance: it is evaluated as the time corresponding to a full melting process of the PCM inside module (with or without the metal foam). The following subsections present the results for each objective.

### 4.1. Effect of PPI Variation

**Figure 6:**
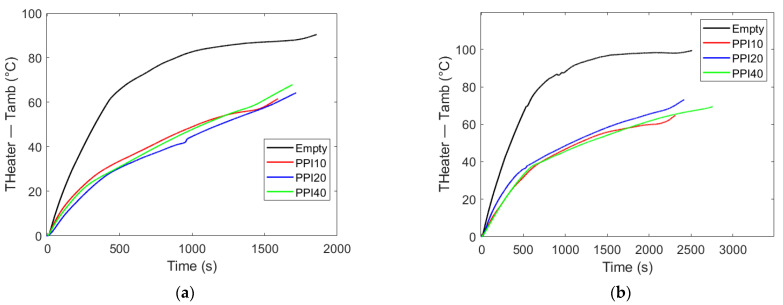
Heater plate temperature above the ambient temperature (°C) vs. time (s). Heater plate temperature evolution for RT55 (**a**) and RT64HC (**b**) under 20 W base heating with PPI10, PPI20, and PPI40 foams. While all foam configurations enhance heat transfer compared to bulk PCM, variations in pore density yield nearly identical temperature profiles due to the constant porosity across foams. This indicates that thermal enhancement is governed primarily by the presence of conductive pathways rather than their linear density (PPI).

**Figure 7:**
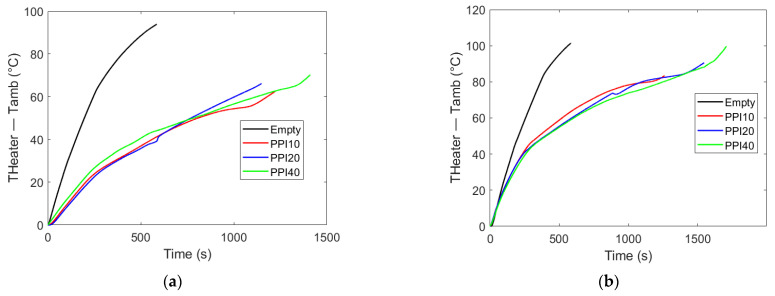
Heater plate temperature above the ambient temperature (°C) vs. time (s). Effect of the varying PPI on PCM melting time. In both cases the heat flux rate is 30 W. (**a**) PCM: RT55; (**b**) PCM: RT64HC.

The influence of pore density (PPI) on the thermal response of PCM–metal foam composites is illustrated in Figure 6 for two representative cases, RT55 (Figure 6a) and RT64HC (Figure 6b), both with a heat flow rate of 20 W, as well as RT55 shown in Figure 7a and RT64HC shown in Figure 7b, both with a heat flow rate of 30 W. In order to avoid any effect of the starting, viz., ambient temperature, the difference between the heated side and ambient temperature is plotted in the vertical axes. Across all experiments, the presence of the metal foam reduced thermal lag (heat transfer delay from one point to another) compared to the control PCM-only case, confirming the beneficial role of conductive pathways in accelerating energy transport from the heater plate to the bulk PCM. However, varying the PPI from 10 to 40 produced only negligible changes in the temperature of the heated side.

This trend suggests that, under constant porosity, the effective thermal conductivity of the composite is governed primarily by the metallic fraction and its connectivity, rather than by the size of pores. These results are consistent with the observations of Diani and Campanale [18], who also found limited sensitivity of melting dynamics to pore density at fixed porosity in side-heated foams.

Although Figure 7 may suggest the PCM-only container outperforms the metal foam composite because the tests finished sooner, this is not the case, as the experiment was halted due to the base temperature reaching the threshold of the silica gel used in fabricating the enclosure.

Overall, these findings highlight that while the integration of metal foams is beneficial, further increasing pore density at constant porosity does not yield proportional thermal improvements, especially under base-heated conditions.

### 4.2. Effect of PCM Variation

**Figure 8:**
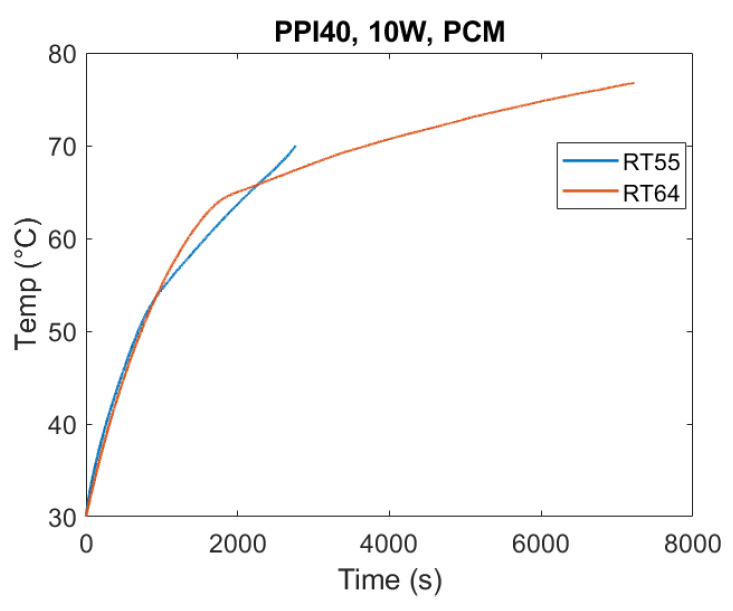
Heater plate temperature evolution for PPI40 under 10 W base heating with RT55 and RT64HC. Melting duration increased with PCM melting temperature and latent heat, with RT64HC requiring the longest time to complete the phase change. These results demonstrate that PCM thermophysical properties—particularly latent heat and heat capacity—strongly govern melting dynamics under identical boundary conditions. In addition, the higher melting temperature of RT64HC led to greater external heat losses, since the apparatus was not thermally insulated.

The effect of PCM type on thermal performance is shown in Figure 8. Under identical test conditions (10 W, PPI40), RT55 exhibited the fastest melting, while RT64HC required the longest time to complete the phase change. This hierarchy reflects their thermophysical properties: RT64HC has the highest latent heat and melting temperature, requiring more energy input to achieve complete transition. For a homogeneous comparison, in the following figures, time is set equal to 0 s when the temperature of the heated base equals 30 °C.

The T-history method confirmed deviations between supplier-reported and measured properties, emphasizing the need for direct experimental characterization in TES studies. Similar conclusions have been drawn in earlier works [20,21,22], where enthalpy–temperature behavior was found to be critical in predicting PCM response under practical operating conditions.

Another key observation was that RT64HC reached higher operating temperatures, which in turn amplified heat losses to the environment due to the lack of thermal insulation in the experimental setup, which, however, replicates a real case scenario.

Overall, these findings highlight a design trade-off: while high-latent-heat and high-melting-temperature PCMs like RT64HC offer superior energy storage capacity, they also exhibit longer melting durations. Having a high melting temperature also makes them more susceptible to thermal losses. Thus, PCM selection must balance energy density requirements with charging/discharging speed depending on the intended application.

**Figure 9:**
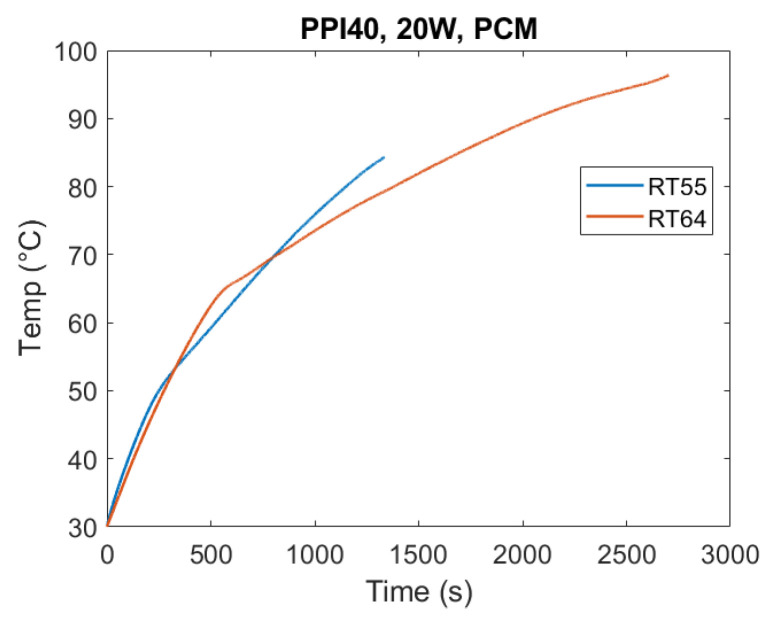
Heater plate temperature evolution for RT55 and RT64HC under 20 W base heating with PPI40. RT64HC completed melting significantly earlier than with 10 W (see Figure 8).

The results also emphasize the combined effect of PCM selection and heat flux level. At low heat flow rate (10 W), conduction dominates and the differences in PCM properties strongly govern melting time. At higher heat flow rates (20 W, see Figure 9), this disparity narrows as greater input energy accelerates melting and partially offsets the slower response of the PCMs having a large latent heat such as RT64HC.

### 4.3. Effect of Heat Flux Variation

**Figure 10:**
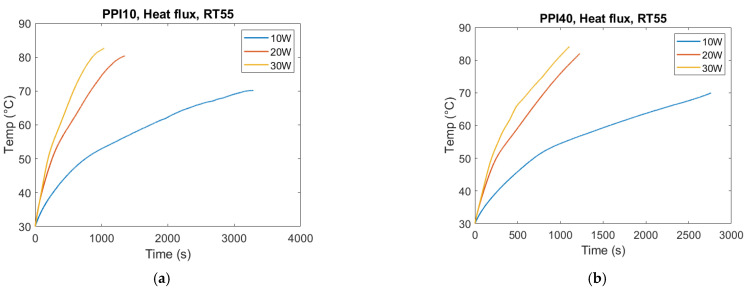
Heater plate temperature evolution for RT55 with PPI10 (**a**) and PPI40 (**b**) under heat flow rates of 10 W, 20 W, and 30 W. Increasing heat flow rate accelerated melting and reduced completion time; however, diminishing returns were observed at higher heat flow rates, with only marginal improvement from 20 W to 30 W. This reflects the combined influence of conduction pathways and external heat losses.

Heat flux was found to be the dominant parameter controlling melting dynamics (Figure 10). Increasing the applied heat flow rate from 10 W to 20 W nearly halved the melting time, but further increasing to 30 W yielded only marginal gains. This diminishing return indicates that once conduction pathways have reached effective utilization, additional input power is partly dissipated through thermal losses rather than accelerating phase change.

Similar saturation trends have been reported in PCM–foam systems [16,17,18], where higher applied heat fluxes led to smaller relative reductions in melting time. In the present study, measured heat losses (3–15% of input) contributed to this effect, particularly at higher fluxes where thermal gradients were larger. Furthermore, base heating geometry ensures relatively uniform energy input, reducing the scope for additional heat flux to enhance local melting rates compared to side heating.

This result underlines the importance of optimizing—not simply maximizing—input flux in PCM-based TES systems. Excessively high flux may increase system complexity and losses without delivering proportional improvements in charging time.

**Figure 11:**
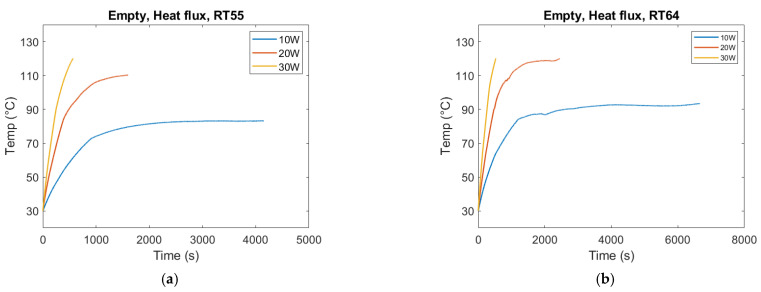
Heater temperature (°C) vs. time (s) at varying heat flow rates for the empty container. (**a**) PCM: RT55. (**b**) PCM: RT64HC.

For the empty container, the same overall effect can be observed. The melting duration reduces as the heat flux increases. In the case of 30 W, the temperature hit the tolerance limit of the silica gel. Therefore, the experiment needed to be stopped at 30 W and the associated trends in Figure 11 do not mean that the PCM has melted.

### 4.4. Results Summary and Data Comparison

Table 4, below, summarizes the experimental results. Some configurations were selected randomly and tested for repeatability, and the same trends were observed for each configuration. It can be observed that there was a general reduction in melting time when metal meshes were used. However, no significant change was observed when meshes with higher PPI were employed. In a few cases, particularly with paraffin RT64HC, meshes with 20 and 40 PPI exhibited similar performance to the case without a metal mesh. This could be due to heat losses to the ambient environment and/or experimental difficulties in identifying the completion of the phase change, since the identification was performed visually. Moreover, as the PPI increased, the heater plate temperature at the end of the melting phase generally decreased. These reductions became less significant with increasing heating power.

The following table, Table 5, shows the reduction in heater plate temperature and the percentage reduction in melting time for the cases with metal meshes compared to the one without.

## 5. Empirical Modeling

The experimental data were compared against the empirical correlation proposed by Diani and Campanale [18], who investigated transient melting of paraffin waxes embedded in aluminum foams under one-dimensional side heating. They derived the following relation between the dimensionless melting temperature *θ* and the combined product of the Fourier and Stefan numbers:

(10) $$\theta =1.9073\times {\left(Fo\times Ste\right)}^{-0.717}$$

where *θ* is the dimensionless melting temperature. According to Diani and Campanale [18], $Fo=\frac{k_{eff}\cdot t_{melt}}{\rho_{eff}\cdot c_{p,eff}\cdot h^{2}}$ is the Fourier number based on effective thermal conductivity *k_eff_*, melting time *t_melt_*, effective density ρ*_eff_*, and specific heat *c_p,eff_*, and *h* is the height of the PCM perpendicular to the melting front. $Ste=\frac{c_{p,eff}\cdot (T_{melt}-T_{i})}{L_{eff}}$ is the Stefan number, representing the ratio of sensible to latent heat, where *T_melt_* and *T_i_* are the melting temperature and the temperature at the beginning of the tests. *L_eff_* is the effective latent heat. Moreover, it is defined as $L_{eff}=\varepsilon \cdot \frac{\rho_{PCM}}{\rho_{eff}}\cdot L_{PCM}$. *L_PCM_* is the latent heat of the PCM. For the evaluation of the results, all the thermophysical properties used were those that were provided by the manufacturer of the paraffins, i.e., Rubitherm.

**Figure 12:**
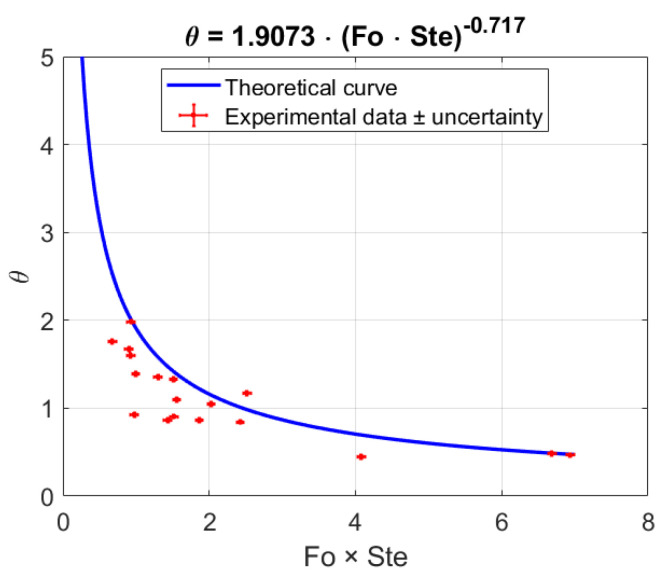
Dimensionless melting temperature (θ) vs. product between Fourier and Stefan number. There was an average uncertainty of ±5% in the parameter θ and of ±2.4% in Fo × Ste, mainly due to the influence of temperature sensors.

Although the original correlation was developed for side-heated foams, in this study it was applied to a base-heated configuration to evaluate its predictive capability under different boundary conditions. This approach was chosen because in [18], the authors tested the same correlation to model the behavior of adiabatic base-heated metal foams reported in [27], and found that it was able to reproduce the general melting trend satisfactorily. Furthermore, due to the presence of the foam structure, the results reported in [18] showed that the melting front was almost parallel to the heater; i.e., no gravity effects were noted. Figure 12 compares the predictions of Equation (10) with the experimentally measured melting dimensionless temperatures, $\theta =\frac{T_{f}-T_{melt}}{T_{melt}-T_{i}}\;$, where *T_f_* is the temperature of the heated plate at the end of the melting process. Meanwhile, the product of Fourier and Stefan numbers is a dimensionless measure of how effectively the supplied heat (via conduction and sensible heating) can overcome the latent heat barrier of the PCM.

Low heat flux conditions are correlated with high values of Fo × Ste and low dimensionless temperatures. This leads to lower temperatures at the heated surface and longer periods needed to fully melt the PCM. Conversely, high dimensionless temperatures and low Fo × Ste values are associated with high heat flux conditions, which result in greater temperatures at the heater–plate contact and shorter melting times.

Overall, the influence of fluid density was not observed. This implies that there is little to no influence of the orientation of the heater on the melting characteristics of PCM embedded in metal foam. In the study, the correlation was verified for the adiabatic base heating model (Mancin et al. [27]). These findings indicate that the orientation of the apparatus is not a major reason behind the disagreement. The disagreement is mostly likely due to heat losses from the system.

Therefore, heat losses were studied from each case to check if they were the reason behind this discrepancy, as Diani and Campanale [18] studied a near-adiabatic system. For the calculation, the conductive (across the polycarbonate plates) and convective (through the ambient) resistances were calculated, by assuming the thermal conductivity to be 1.1 W/(m K) and convection coefficient to be 5 W/(m^2^ K). The width of the polycarbonate sheet was 5 mm. The heat loss was calculated for all four sides covered with the polycarbonate sheet, and for the top only the convective heat loss was considered as it was directly in contact with the ambient air. For the losses, the mid-point temperature was considered as the reference maximum temperature. It was assumed that all the losses proceeded from the center of the structure to the ambient air. In the case of base heating, the following table, Table 6, presents the heat losses for all individual experiments.

The losses in some cases were higher than 3–5%; i.e., the near-adiabatic conditions in the study performed by Diani and Campanale [18] are not reached. This resulted in the disagreement between the model and experiment being particularly large, especially in cases where heat losses exceeded 10%.

Moreover, the data could not be corrected for the heat losses, as doing so was not feasible, due to multiple factors affecting the heat loss from the system. Plus, using one value, i.e., the average percentage heat loss, to correct all the data points was also not feasible, because the data were very spread out. The following table, Table 7, shows the mean heat loss, standard deviation, and maximum values for the cases that displayed the highest deviations from the model.

## 6. Conclusions

This study experimentally investigated the melting dynamics of PCM–metal foam composites under base heating, examining the roles of pore density, PCM type (melting temperature), and heat flux. The T-history method was used to accurately determine the thermophysical properties of RT42, RT55 and RT64HC, providing more reliable data than supplier datasheets.

The results demonstrated that embedding PCMs (RT55 and RT64HC) within metallic foams improves thermal uniformity and reduces thermal lag compared to bulk PCM. Variations in pore density between 10, 20, and 40 PPI had negligible influence on melting completion time under constant porosity, suggesting that the dominant effect is the presence of a conductive pathway rather than its pore density. PCM melting temperature strongly affects melting duration, with higher latent heat materials requiring longer times to fully melt. Heat flux was found to be the dominant parameter, with higher fluxes significantly reducing melting times, although diminishing returns appeared at elevated levels.

The empirical correlation proposed by Diani and Campanale [18] for side-heated foams was applied to the present base-heated configuration. While the model captured the overall melting trends, it consistently underpredicted the melting completion times at higher heat fluxes. This deviation arises from two key factors: (i) a difference in heat transfer geometry between side heating and base heating, and (ii) unavoidable external heat losses (approximately 3–15% of the input power) due to the uninsulated experimental setup. Among these, heat losses were found to play the more dominant role. Notably, the original validation of the correlation against an idealized adiabatic, one-dimensional base-heating case [27] highlights the importance of developing refined models that explicitly account for non-adiabatic base-heating conditions.

Overall, PCM–metal foam composites were shown to significantly enhance heat transfer and reduce thermal lag, confirming their potential in compact TES modules, electronics-cooling, and renewable energy applications. Future research should prioritize (i) the design of additively manufactured metallic architectures with optimized pore geometries, (ii) developing a new correlation explicitly for base-heated configurations, and (iii) systematic evaluation of long-term cycling performance to accelerate the practical deployment of PCM–metal composites in advanced thermal management systems.

## Figures and Tables

**Table 1:** Legend for the symbols used in Figure 1.

Symbol	Meaning
*T*_0_	Initial homogeneous temperature
*T_m_*_1_	Temperature at which phase change starts
*T_m_*_2_	Temperature at which phase change ends
*T_r_*	Common end temperature (Water and PCM)

**Table 2:** Results of the T-history method.

Thermophysical Properties	Experimental Results			Values Defined in the Rubitherm Datasheet		
	RT42	RT55	RT64HC	RT42	RT55	RT64HC
*c_ps_* [kJ/(kg K)]	3.316	2.259	1.335	2	2	2
*c_pl_* [kJ/(kg K)]	2.573	2.664	2.448	2	2	2
*H_l_* [kJ/kg]	165.0	182.4	242.7	165	170	250

**Table 3:** Properties of metal foams.

Name	Linear Porosity (PPI) *	Volumetric Porosity *	Surface Area per Unit Volume * (m^−1^)	Fiber Thickness ** (mm)	Fiber Length ** (mm)
Al-10-6.8	10	93.2%	692	0.45	1.785
Al-20-6.8	20	93.2%	1156	0.367	1.218
Al-40-7.0	40	93.0%	1679	0.324	1.072

* measured and provided by the manufacturer. ** measured by Mancin et al. [26].

**Table 4:** Summary of the observed trends for paraffins RT55 and RT64HC.

	RT55				RT64HC	
	Empty					
Heat flow rate (W)	10	20	30	10	20	30
*T_heater_* (°C) at phase change completion	81.15	113.1	120.11	93.45	119.95	120
Phase change completion time (s)	4526	1858	586 *	6749	2412	585 *
	PPI 10					
Heat flow rate (W)	10	20	30	10	20	30
*T_heater_* (°C) at phase change completion	78.76	85.79	89.87	81.89	94.73	94.02
Phase change completion time (s)	4229	1592	1222	5704	2042	2313
	PPI 20					
Heat flow rate (W)	10	20	30	10	20	30
*T_heater_* (°C) at phase change completion	70.661	86.643	91.44	85.41	101.4	111.4
Phase change completion time (s)	3144	1727	1149	6721	2429	1546
	PPI 40					
Heat flow rate (W)	10	20	30	10	20	30
*T_heater_* (°C) at phase change completion	69.97	95.06	93.26	82.67	96.37	120
Phase change completion time (s)	2739	1702	1311	9721	2684	1557

* Did not finish the phase change process. The experiment was stopped as the temperature of the heater reached 120 °C, i.e., the limit for the silica gel used to make the enclosure.

**Table 5:** Effect of using metal meshes for the paraffin RT55.

Metal Mesh	Heat Flow Rate (W)	Reduction in the Temperature of the Heated Plate (K)	% Reduction in Melting Time
	10	2.4	6.6
PPI10	20	27.3	14.3
	30	30.2	N/A *
	10	10.5	30.5
PPI20	20	26.5	7.1
	30	28.7	N/A *
	10	11.2	39.5
PPI30	20	18.1	8.4
	30	26.9	N/A *

* cannot compare with the empty container case, as the phase change did not complete in that case.

**Table 6:** Results of the heat loss calculations for PCMs RT55 and RT64HC.

Heat Flow Rate	PPI	PCM (Paraffin)	AVG Loss [%]
10 W	10 PPI	RT55	9.89
20 W	10 PPI	RT55	4.04
30 W	10 PPI	RT55	2.95
10 W	20 PPI	RT55	6.13
20 W	20 PPI	RT55	3.25
30 W	20 PPI	RT55	1.85
10 W	40 PPI	RT55	9.09
20 W	40 PPI	RT55	5.10
30 W	40 PPI	RT55	3.07
10 W	10 PPI	RT64HC	14.70
20 W	10 PPI	RT64HC	5.62
30 W	10 PPI	RT64HC	5.03
10 W	20 PPI	RT64HC	11.09
20 W	20 PPI	RT64HC	5.19
30 W	20 PPI	RT64HC	3.45
10 W	40 PPI	RT64HC	13.32
20 W	40 PPI	RT64HC	6.04
30 W	40 PPI	RT64HC	4.44

**Table 7:** Statistical parameters for the cases with the worst deviations.

Case	Mean Loss (%)	Standard Deviation (%)	Maximum Value (%)
10W/40PPI/RT55	3.25	1.90	7.19
10W/10PPI/RT64	14.70	4.60	20.90
20W/10PPI/RT64	5.62	2.74	10.98
30W/10PPI/RT64	5.03	2.70	9.35

## Data Availability

The original contributions presented in this study are included in the article. Further inquiries can be directed to the corresponding author.
